# Circulating neuropeptide Y dynamics and performance during exercise in heart failure patients with contemporary medical and device therapy

**DOI:** 10.1113/EP092325

**Published:** 2025-01-24

**Authors:** Thamali Ayagama, Peregrine G. Green, Cheryl Tan, Cristiana Monteiro, David A. Holdsworth, Neil Herring

**Affiliations:** ^1^ Burdon Sanderson Cardiac Science Centre, Department of Physiology, Anatomy and Genetics University of Oxford Oxford UK; ^2^ Division of Cardiovascular Medicine, Radcliffe Department of Medicine University of Oxford Oxford UK; ^3^ Oxford Heart Centre, John Radcliffe Hospital University of Oxford NHS Foundation Trust Oxford UK; ^4^ Ludwig Institute for Cancer Research, Nuffield Department of Medicine University of Oxford Oxford UK

**Keywords:** cardiopulmonary exercise testing, congestive heart failure, neuropeptide Y, sympathetic cotransmitter

## Abstract

High cardiac sympathetic drive and release of the sympathetic cotransmitter neuropeptide Y (NPY) are significant features of congestive heart failure (CHF), in which resting venous NPY levels are known to be associated with mortality. However, whether circulating NPY levels increase during exercise in CHF when they are already elevated is controversial. We sought to establish the dynamics of circulating NPY levels in CHF patients treated with contemporary medical therapy and devices in relationship to indices of performance linked to long‐term prognosis. CHF patients (*n* = 15) underwent cardiopulmonary exercise testing with venous blood sampling at rest, peak exercise and recovery. These patients had significantly higher resting venous NPY levels compared with an age‐ and sex‐matched control group of patients (*n* = 16) with normal left ventricular function (40 ± 6.9 vs. 9.0 ± 4.6 pg/mL, respectively; *P *< 0.0001). In CHF patients, NPY levels increased significantly from baseline to peak exercise (to 93.5 ± 42.1 pg/mL; *P *= 0.0004) and remained elevated during recovery (86.8 ± 44.6 pg/mL; *P *= 0.0018). The peak (*r* = 0.58, *P *= 0.0222) and recovery (*r* = 0.56, *P *= 0.0304) NPY levels and the ability to increase NPY from baseline (*r* = 0.53, *P *= 0.0427) showed significant positive correlations with heart rate recovery at 1 min, but not with peak oxygen consumption. In CHF patients, the ability to increase NPY levels on exertion is correlated with heart rate recovery, a known prognostic indicator for mortality. These findings suggest that NPY dynamics during exercise might provide valuable insights into sympathetic responses and prognosis in CHF patients.

## Abstract (highlights)

- **What is the central question of this study?**
  We sought to establish dynamics of circulating neuropeptide Y (NPY) levels in congestive heart failure (CHF) patients treated with contemporary medical therapy and devices in relationship to indices of performance linked to long‐term prognosis.
- **What is the main finding and its importance?**
  Peripheral venous NPY at rest in CHF patients was several fold higher compared with a well‐matched control cohort. Peripheral venous NPY levels increased significantly further from rest to peak exercise in heart failure. Peak and recovery NPY levels and the ability to increase NPY during exercise from baseline were all significantly correlated with heart rate recovery at 1 min, a strong prognostic marker for mortality in this cohort.

## 1 INTRODUCTION

Congestive cardiac failure (CHF) is associated with high resting sympathetic drive, leading to increased release of noradrenaline (Cohn et al., 1984) and the cotransmitter neuropeptide Y (NPY) (Ajijola et al., 2020; Hulting et al., 1990; Maisel et al., 1989; McDowell et al., 2024), in addition to vagal withdrawal (La Rovere et al., 1998; Nolan et al., 1998), which are indicators of poor prognosis for morbidity and mortality (Herring, Kalla et al., 2019). Although catecholamine and NPY release are known to increase during exercise in healthy patients (Eugster et al., 2022; Kaijser et al., 1990; Lacroix et al., 1997; Lind et al., 1994; Morris et al., 1986), whether NPY levels can increase significantly in CHF when they are already elevated is controversial. Early studies found little change in mean peripheral venous NPY concentration during exercise (Maisel et al., 1989; Nicholls et al., 1992), although one study did identify a small rise (Ullman et al., 1994). However, these studies were limited to using non‐specific assays and lack of contemporary pharmacological and device treatments for heart failure.

In the CHF population, quality of life and exercise capacity are inextricably linked, and cardiopulmonary exercise testing (CPET) is the gold‐standard quantitative method to assess this (Guazzi et al., 2012; Malhotra et al., 2016). Left ventricular ejection fraction (Cohn et al., 1993), peak exercise oxygen consumption (${\dot{V}}_{O_{2}\mathrm{peak}}$) (Keteyian et al., 2016) and heart rate recovery (HRR) following exercise (Cole et al., 1999) are strong prognostic markers for mortality in this population. NPY released during high‐level cardiac sympathetic stimulation can act on cardiomyocyte Y1 receptors to facilitate calcium entry and augment contraction, even in the presence of β‐blockade (Kalla et al., 2020). However, it can also cause coronary vasoconstriction via vascular smooth muscle Y1 receptors (Cuculi et al., 2013; Gibbs et al., 2022; Herring, Tapoulal et al., 2019), which might limit ${\dot{V}}_{O_{2}\mathrm{peak}}$. NPY can also act on Y2 receptors in cardiac postganglionic cholinergic ganglia to reduce acetylcholine release and bradycardia (Herring et al., 2012, 2008). Given that HRR is mediated primarily by vagal rebound, the NPY release profile during exercise and recovery might play an important role.

We therefore sought to characterize the dynamics of NPY release during exercise in CHF patients undertaking CPET treated with optimized modern medical therapy and cardiac resynchronization therapy (CRT) and assess whether the NPY release profile was correlated with key prognostic variables, such as ${\dot{V}}_{O_{2}\mathrm{peak}}$ and HRR.

## 2 MATERIALS AND METHODS

### 2.1 Ethical approval

The study was approved by the local research ethics committee (REC 18/SC/0611, 10/H0408/24 and 10/H0606/36) and the institutional review board committee (OUH PID 13808). Written informed consent was collected from all the participants, and the study complied with the *Declaration of Helsinki*. The study was registered on ClinicalTrials.gov (NCT03768804).

### 2.2 Participants

Patients with a CRT device implanted according to current European Society of Cardiology guidelines (Glikson et al., 2021) and being followed up by Oxford University Hospitals National Health Service Foundation Trust were recruited. Inclusion criteria included age ≥18 years and the capability to provide informed consent, ability to exercise to perform CPET, and having a CRT device implanted for ≥6 months. Exclusion criteria included atrial arrhythmia, pregnancy or breastfeeding, ≥80% atrial pacing or chronotropic incompetence (defined as the use of a rate‐response algorithm), and any concurrent contraindicating conditions for the use of CPET (Green et al., 2024). CRT patients were compared with a control group undergoing elective coronary angiography who were found to have unobstructed coronary arteries and normal left ventricular systolic function on transthoracic echocardiography, recruited as part of the Oxford Acute Myocardial Infarction (OxAMI) study.

### 2.3 Cardiopulmonary exercise testing

The CRT devices were programmed with fixed atrioventricular delays of 120/120 ms but otherwise optimized using the Oxford method to produce the narrowest QRS duration of the best morphology, as previously described (Robertson et al., 2022). Participants underwent CPET using a static bicycle ergometer (Ergoselect™, Ergoline, Germany) and a spiroergometry system (Metalyzer™ 3B, Cortex Medical, Germany), in conjunction with an oro‐nasal V2 reusable facemask (Hans Rudolph, Shawnee, KS, USA). Prior to each test, the spiroergometry system was calibrated to known oxygen and carbon dioxide concentrations and atmospheric pressure. A 15 W step protocol with 3 min stages and a 15 min recovery period was used. Twelve simultaneous lead ECG recordings were obtained using commercial software (Custo Med, Germany), and the data were recorded using commercial software (MetaSoft™ Studio v.5.13.0, Cortex Medical). CPET data were smoothed using a rolling average of breath‐by‐breath data based on a 10 s rolling period. The ${\dot{V}}_{O_{2}\mathrm{peak}}$ was defined as the maximum oxygen uptake achieved over a rolling 20 s average.

The Borg rating of perceived exertion was obtained from participants at the end of each stage, and CPET continued until the participant could not maintain the escalating workload at a pedalling cadence of ≥60 r.p.m. Venous blood was withdrawn from a peripheral cannula at baseline, at peak exercise and after 15 min of recovery to measure NPY concentration.

### 2.4 Enzyme‐linked immunosorbent assay

Blood samples were centrifuged and immediately frozen and stored at −80°C until the time of assay. ELISA (EZHNPY‐25K, Millipore) was used to measure the concentration of NPY in 50 µL of sample in duplicates against serially diluted protein standards, as described previously (Ajijola et al., 2020; Cuculi et al., 2013; Gibbs et al., 2022; Herring, Tapoulal et al., 2019; McDowell et al., 2024). The assay has a limit of detection of 2–3 pg/mL, and there is 0% cross‐reactivity with structurally similar peptides (such as peptide YY, pancreatic polypeptide, gastric inhibitory polypeptide, ghrelin, proinsulin or glucagon), The *R* ^2^ value for calibration is >0.95, and inter‐ and intra‐assay coefficients of variation are <8.1% and <6.1%, respectively.

### 2.5 Statistical analysis

Continuous data were assessed using a normality test (Shapiro–Wilk) and presented as the mean ± SD or median [interquartile range], as appropriate. Discrete data were presented as ‘*n*’ or a percentage. Between‐group analysis was performed using Student's unpaired *t*‐test with Welch's correction for normally distributed data or using Fisher's exact test for contingency tables. Normally distributed paired data at different time points were analysed using a repeated‐measurements one‐way ANOVA and Dunnett's multiple comparisons test. Pearson's correlation coefficient was used to measure linear correlation between two normally distributed variables. The statistical significance threshold was considered at a *P*‐value of ≤0.05. All the statistical analyses were performed with Graphpad Prism v.9 (Graphpad Holdings, CA, USA).

## 3 RESULTS

### 3.1 Baseline clinical characteristics

**FIGURE 1:**
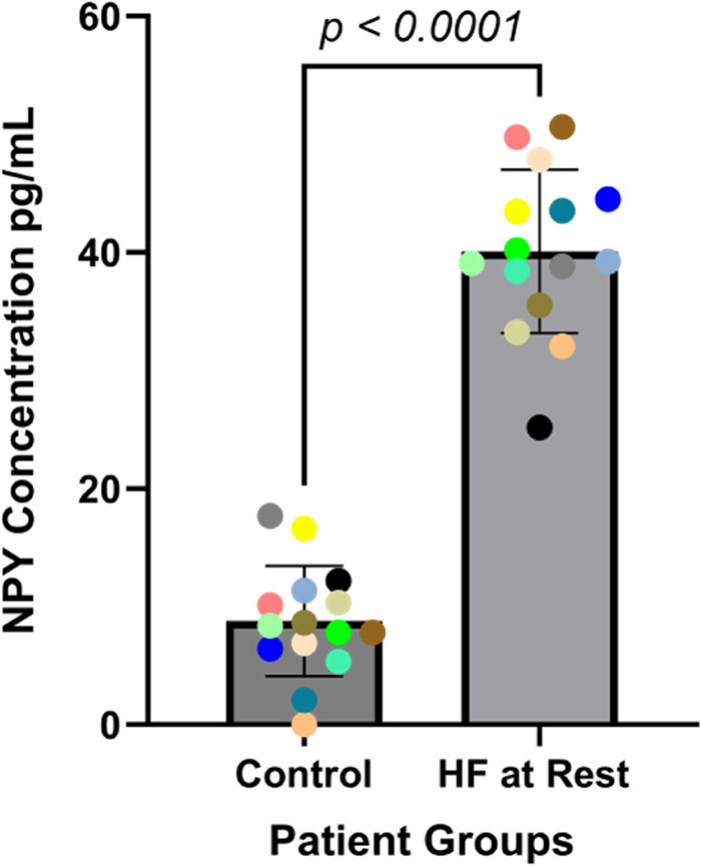
Resting NPY levels in control and HF patients. The HF patients (*n* = 15) had significantly higher resting peripheral venous NPY levels (40 ± 6.9 pg/mL; *P *< 0.0001) compared with the age‐ and sex‐matched control group of patients (*n* = 16, 9.0 ± 4.6 pg/mL). Abbreviations: HF, heart failure; NPY, neuropeptide Y.

Fifteen CHF patients were recruited, and baseline demographics and clinical details are given in Table 1. These patients had undergone significant QRS duration narrowing with CRT (from 164 ± 21 to 129 ± 18 ms; *P *< 0.0001) and significant reverse remodelling at 6 months, with reduction in left ventricular internal diameter in end diastole (from 58 ± 8 to 48 ± 8 mm; *P *= 0.005) and improvement in systolic function, although this still remained impaired (left ventricular ejection fraction from 29% ± 7% to 44% ± 7%; *P *< 0.0001). These patients were compared with 16 patients with normal left ventricular systolic function and unobstructed coronary arteries recruited to the OxAMI study who were well matched in terms of age and sex, as shown in Table 1. Resting NPY levels were significantly higher in the CHF group (40 ± 6.9 compared with 9.0 ± 4.6 pg/mL; *P *< 0.0001), as shown in Figure 1.

**TABLE 1:** Patient demographics.

Parameter	Control (*n* = 16)	Heart failure (*n* = 15)	*P*‐value
Age (years)	67 ± 3	70 ± 10	0.44
Males [*n* (%)]	10/16 (63)	9/15 (60)	1.00
Background [*n* (%)] Diabetes mellitus Ischaemic heart disease Non‐ischaemic cardiomyopathy	3/16 (19) 0 0	6/15 (40) 4/15 (27) 11/15 (73)	0.25 0.04 <0.0001
Medications [*n* (%)] β‐Blockers ACEi/ATRA/ARNi MRA SGLT2i	10/16 (63) 11/16 (69) 0 0	15/15 (100) 14/15 (93) 10/15 (67) 3/15 (20)	0.02 0.17 <0.0001 0.10
Blood pressure and heart rate Systolic (mmHg) Diastolic (mmHg) Heart rate (beats/min)	130.3 ± 27.8 64.1 ± 10.2 66.4 ± 14.4	129.7 ± 19.9 81.7 ± 12.8 74.7 ± 12.7	0.95 <0.001 0.11
Echocardiography Ejection fraction (%) LVIDd (mm)	63 ± 8 48 ± 8	44 ± 7 48 ± 8	<0.0001 0.95

Abbreviations: ACEi, angiotensin‐converting enzyme inhibitor; ARNi, angiotensin receptor/neprilysin inhibitor; ATRA, angiotensin II receptor antagonist; LVIDd, left ventricular internal diameter in end diastole; MRA, mineralocorticoid receptor antagonist; SGLT2i, sodium/glucose cotransporter 2 inhibitor.

### 3.2 Performance characteristics of CHF patients during CPET

The CHF patients at the time of CPET were New York Heart Association class 2 [1–2] and able to exercise up to a Borg rating of perceived exertion of 19 [17–19], reaching 79% ± 12% of their age‐predicted heart rate maximum. The performance characteristics of the CPET are summarized in Table 2.

**TABLE 2:** Cardiopulmonary exercise testing performance parameters of 15 patients with congestive cardiac failure.

Performance characteristic	Performance level
Heart rate at rest (beats/min)	74 [68–83]
Heart rate at peak exercise (beats/min)	116.5 [101.8–142.3]
Heart rate recovery (beats/min)	9.89 [6.78–17.2]
${\dot{V}}_{O_{2}\mathrm{peak}}$ (mL/kg/min)	14.93 [13.61–18.35]
Time to ${\dot{V}}_{O_{2}\mathrm{peak}}$ (s)	1294 [1157–1647]
Work rate at peak (W)	90 [75–120]
Respiratory exchange ratio at peak	1.02 [0.99–1.11]

Abbreviation: ${\dot{V}}_{O_{2}\mathrm{peak}}$, peak exercise oxygen consumption.

### 3.3 NPY dynamics during exercise

**FIGURE 2:**
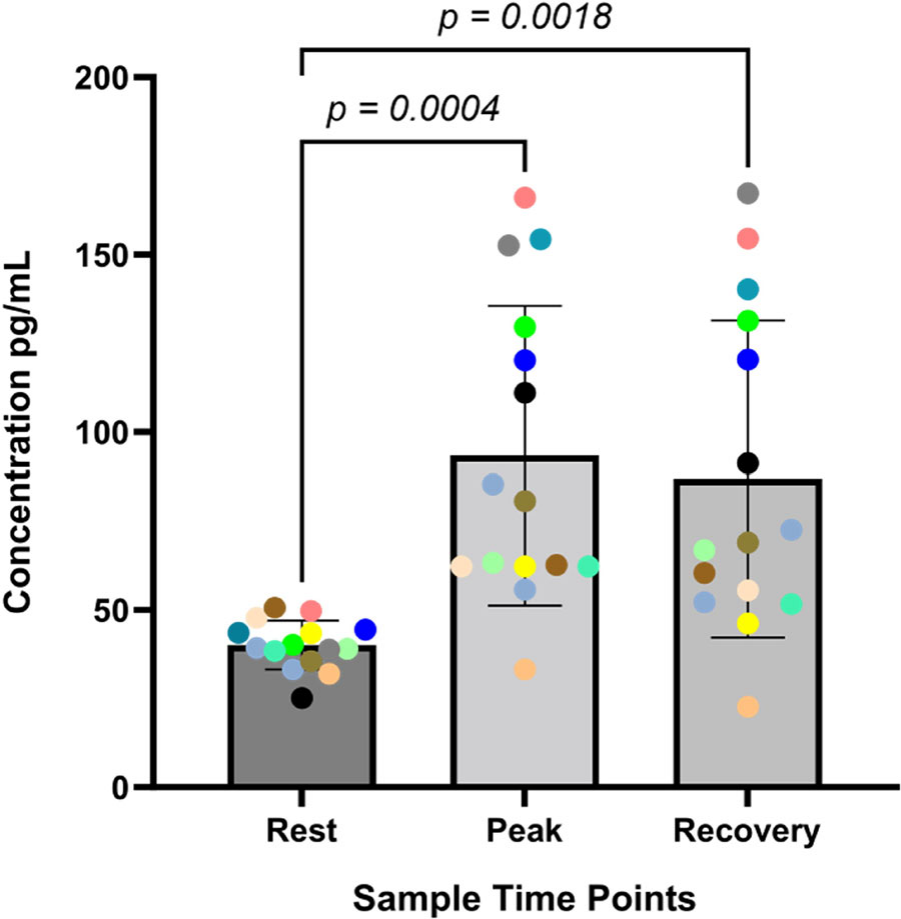
Dynamics of NPY during cardiopulmonary exercise testing in patients with congestive heart failure. Peripheral venous NPY levels were increased significantly from baseline to peak exercise (from 40.08 ± 6.90 to 93.46 ± 42.13 pg/mL; *P *= 0.0004) and remained elevated after 15 min of recovery (to 86.84 ± 44.60 pg/mL; *P *= 0.0018) in 15 heart failure patients. Abbreviation: NPY, neuropeptide Y.

**FIGURE 3:**
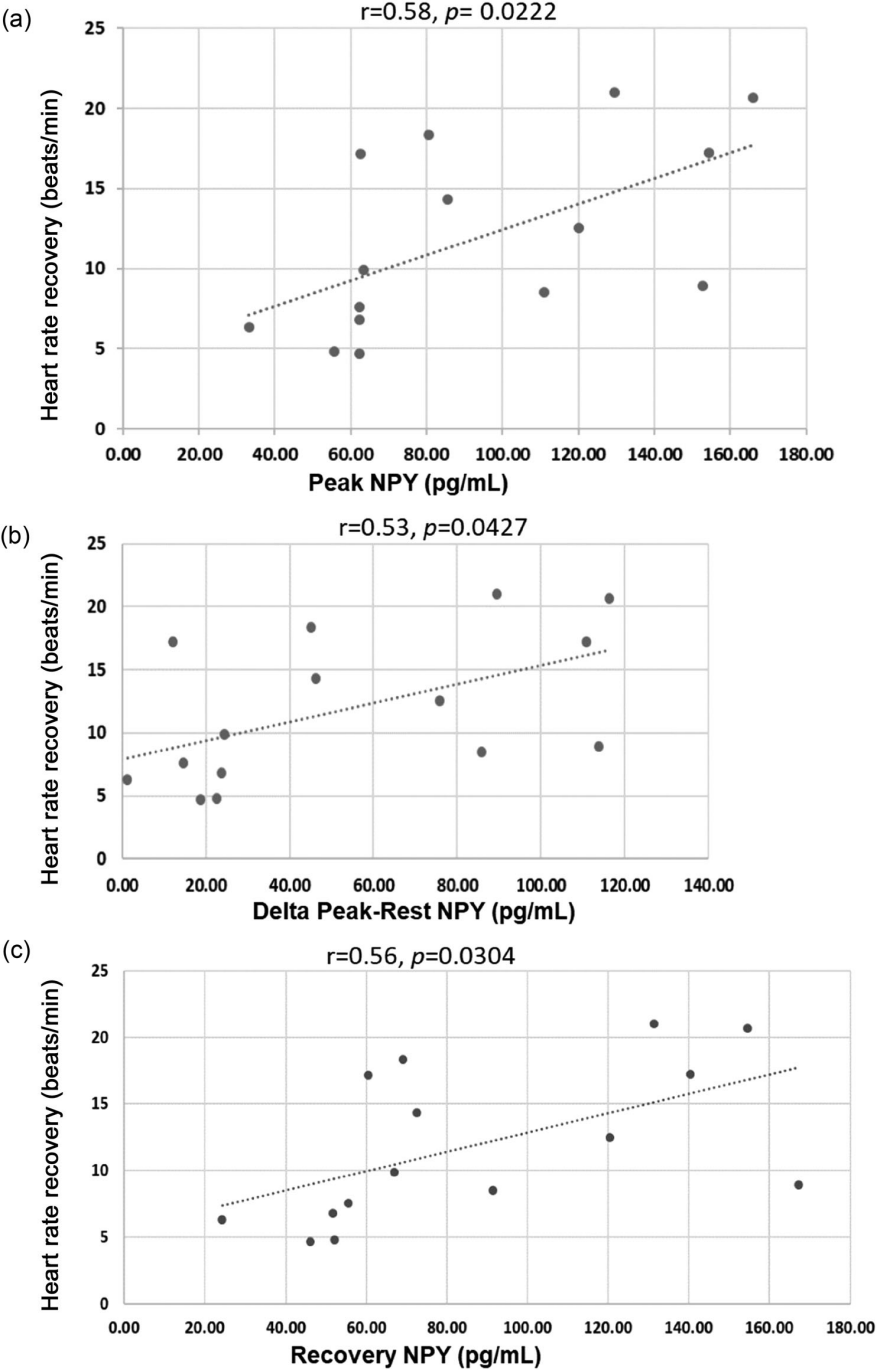
Correlation of peripheral venous NPY levels with heart rate recovery after cardiopulmonary exercise testing. The peak peripheral venous NPY level (*r* = 0.58, *P* = 0.0222; a), the ability to increase peripheral venous NPY from baseline (*r* = 0.53, *P* = 0.0427; b) and the peripheral venous NPY level after 15 min recovery (*r* = 0.56, *P* = 0.0304; c) were significantly correlated with heart rate recovery at 1 min in 15 patients with congestive heart failure. Abbreviation: NPY, neuropeptide Y.

Peripheral venous NPY levels increased significantly from rest to peak exercise but remained significantly elevated after 15 min of recovery, as shown in Figure 2. The NPY concentrations at rest (*r* = 0.18, *P *= 0.52), peak exercise (*r* = 0.42, *P* = 0.12) or recovery (*r* = 0.40, *P *= 0.14) time points were not correlated with ${\dot{V}}_{O_{2}\mathrm{peak}}$ or the peak work rate achieved (rest, *r* = −0.50, *P* = 0.06; peak, *r* = 0.42, *P *= 0.12; recovery, *r* = 0.38, *P *= 0.16). However, the peak NPY levels, the ability to increase NPY during exercise from baseline and the recovery NPY level were all significantly correlated with HRR at 1 min, as shown in Figure 3.

## 4 DISCUSSION

The main findings of this study are as follows. Firstly, resting peripheral venous levels of NPY are significantly elevated in CHF patients treated with CRT and contemporary medical therapy compared with age‐ and sex‐matched control subjects with normal left ventricular systolic function. Secondly, this group of CHF patients can significantly increase their peripheral venous NPY levels during maximal exercise, and these levels remain elevated even after 15 min of recovery. Thirdly, peak NPY levels and the ability to increase NPY levels during exercise were positively correlated with HRR, a known prognostic indicator for reduced mortality in the heart failure population.

### 4.1 Elevated resting NPY levels in CHF

We found a ∼4‐fold higher peripheral venous NPY level at rest in a cohort of heart failure patients treated with contemporary guideline‐directed medical and device therapy, in comparison to a well‐matched cohort in terms of age, sex and left ventricular internal diameter in end diastole (40 ± 6.9 vs. 9.0 ± 4.6 pg/mL, respectively). Others have reported peripheral venous NPY levels using an assay with a similar level of detection to ours of ∼2 pg/mL in healthy normal patients, although this was a younger cohort (20–55 years old) (Grouzmann et al., 1989). This greatly strengthens previous assumptions that NPY levels are indeed elevated in heart failure.

We have reported peripheral venous levels of NPY 25.8 ± 18.2 pg/mL in 833 heart failure patients of similar age and sex with a range of ejection fractions (30% having heart failure with a preserved ejection fraction; McDowell et al., 2024). Here, NPY levels were not correlated with ejection fraction or CRT use, although peripheral venous NPY levels of >29 pg/mL were associated with cardiovascular and all‐cause mortality even when adjusted for other prognostic variables, including brain typenatriuretic peptide (BNP). We have found coronary sinus levels in patients with severely impaired LVSF undergoing CRT implant to be higher still at 85.1 ± 3.0 pg/mL, with levels of <130 pg/mL associated with event‐free (death, cardiac transplant or left ventricular assist device) survival (Ajijola et al., 2020). Likewise, 163 patients of a similar age and sex to those in the present study had peripheral venous NPY levels of 28.6 ± 2.2 pg/mL, immediately after primary percutaneous coronary intervention for ST‐elevation myocardial infarction, where levels of >21.4 pg/mL were correlated with the risk of heart failure or mortality (Gibbs et al., 2022). Blood sampling in the present study was undertaken in the CHF group immediately before the start of CPET, and there could be some feedforward central command already increasing sympathetic drive on anticipation of exercise (Basnayake et al., 2012), which we cannot discount. However, this is also likely to have occurred in the control group undergoing the stress of an elective coronary angiogram.

### 4.2 NPY dynamics during exercise in CHF

Peripheral venous NPY levels in our CHF group more than doubled at peak exercise despite being significantly elevated at rest. Studies using less specific assays, prior to the advent of modern heart failure therapies, have found no increase (Maisel et al., 1989; Nicholls et al., 1992) or a small increase (Ullman et al., 1994) in peripheral venous NPY concentrations during exercise.

In comparison, in a population of 51 young healthy patients (32 male, age 23.5 ± 3.7 years, ejection fraction 63.3% ± 5%) undergoing CPET at our institution, NPY levels increased >10‐fold to a significantly higher peak (197 ± 140 pg/mL), then fell significantly during recovery (89 ± 53 pg/mL). Our assay, unlike recently published liquid chromatography–mass spectrometry approaches, is unable to distinguish NPY fragments (for example, NPY3–36) and proNPY levels (Vocat et al., 2020), which have also been described to increase in young healthy volunteers during exercise, with NPY3–36 rising during recovery (Eugster et al., 2022). Liquid chromatography–mass spectrometry is not readily available clinically; instead, we used the same ELISA that has been used in several large‐scale clinical studies. We speculate that given that NPY levels can also increase in heart failure patients, a similar delayed rise in NPY3–36 during recovery would also be seen. In young healthy patients, inhibition of dipeptidyl‐peptidase‐4, which cleaves NPY1–36 to NPY3–36, increases NPY1–36 levels and improves the time to exhaustion during CPET, although it does not change ${\dot{V}}_{O_{2}\mathrm{peak}}$ (Bourdillon et al., 2022). Truncation of NPY1–36 to NPY3–36 results in loss of its affinity for the Y1 receptor, whilst Y2 and Y5 receptor affinity remain. Y1 receptor signalling is associated with cardiomyocyte calcium handling, positive inotropy, hypertrophy and vasoconstriction, whilst Y2 and Y5 receptor signalling are also associated with angiogenesis, improved cell survival and reduced fibrosis (Bussmann et al., 2025). It should be noted that dipeptidyl‐peptidase‐4 inhibitors, used chronically to treat type 2 diabetes mellitus, can cause adverse heart failure events and a higher risk of cardiovascular hospitalization and death (Packer, 2018). We also speculate that a delayed and sustained rise in NPY3–36 following cessation of exercise might therefore be a poor prognostic indicator, although this is yet to be established.

### 4.3 NPY and heart rate recovery after exercise

In our CHF cohort, peak NPY and the ability to increase NPY from baseline, in addition to recovery NPY levels, were all positively correlated with HRR. It is worth noting that in the young healthy cohort from our institution mentioned earlier, this observation was not seen, but rather peak NPY levels were positively correlated with peak oxygen consumption (*r* = 0.35, *P *= 0.006) and peak work rate (in watts; *r* = 0.43, *P *= 0.001), which was not seen in the CHF cohort. It appears that healthy and/or younger patients have significantly higher ‘sympathetic reserve’, which is correlated with the level of performance. This might be lost during CHF, in which baseline sympathetic drive is high, and patients are treated with β‐blockers to reduce mortality. Moreover, NPY levels remain high following exercise. The better the sympathetic reserve seen in CHF patients, the better their HRR. A limitation to our study is the lack of an age‐ and sex‐matched control group with known normal left ventricular function, similar comorbidities, body mass index and cardiovascular fitness who underwent the same CPET protocol. Recruiting such a control group would be challenging, and these patients would not be on prognostic heart failure medications, which would be a major confounder. For example, both β‐blockers and reducing the chronotropic response to exercise can prevent the stimulation of NPY release by catecholamines (van Weperen et al., 2025). However, we do not feel that a lack of a control group diminishes the new and clinically relevant finding that HRR, which is strongly associated with prognosis in heart failure, is correlated with NPY levels during CPET, which is an important finding in its own right.

The HRR is an easily derived independent prognostic marker of mortality (Cole et al., 1999; Shetler et al., 2001), mediated by vagal rebound in the presence of high sympathetic drive (Gourine & Ackland, 2019). It is therefore a marker of increased vagal tone in relationship to cardiovascular fitness with exercise training, in addition to vagal withdrawal in cardiac pathology such as myocardial infarction and heart failure (Arai et al., 1989; Imai et al., 1994). The mechanistic basis for the beneficial effects of efferent parasympathetic drive in heart failure are well documented and include reducing heart rate, increasing coronary perfusion time, preventing ventricular arrhythmias (Kalla et al., 2016) and anti‐inflammatory effects to prevent adverse remodelling both directly and indirectly through antagonizing cardiac sympathetic drive (Herring, Kalla et al., 2019).

Neuropeptide Y can inhibit acetylcholine release from postganglionic parasympathetic neurons and vagal bradycardia in the presence of β‐blockade (Herring et al., 2012, 2008). This provides a mechanistic explanation for the observation in heart failure patients regarding HRR reported here. Those CHF patients who can increase and decrease their NPY levels dynamically, as seen in young healthy patients, rather than having a persistently high and flat NPY release profile, might therefore experience less peripheral vagal inhibition. The association between NPY level and HRR in CHF might also reflect greater centrally mediated preganglionic vagal rebound (Machhada et al., 2020) or, alternatively, simply be an indirect marker of overall cardiovascular fitness. NPY might also have a role beyond that of being simply a sympathetic cotransmitter, given that recent tissue‐clearing work in pig and human hearts demonstrates NPY expression in cholinergic ganglia in the right atrial ganglionic plexi around the sinoatrial node (Hanna et al., 2021). The functional role of this source of NPY and whether it changes in CHF are unknown.

## 5 CONCLUSION

We have shown that peripheral venous levels of the sympathetic cotransmitter NPY are significantly increased during exercise in patients with CHF despite having significantly higher resting levels compared with age‐ and sex‐matched controls. CHF patients with improved sympathetic reserve in terms of their ability to change vagolytic NPY levels dynamically have better HRR on cessation of exercise, a known prognostic indicator for mortality.

## AUTHOR CONTRIBUTIONS

Neil Herring: conception and design. Thamali Ayagama, Peregrine G. Green, Cheryl Tan, Cristiana Monteiro, David A. Holdsworth and Neil Herring: acquisition, analysis or interpretation of data. Thamali Ayagama: drafting the work. Peregrine G. Green, Cheryl Tan, Cristiana Monteiro, David A. Holdsworth, Neil Herring: revising the manuscript. All authors approved the final version of the manuscript and agree to be accountable for all aspects of the work in ensuring that questions related to the accuracy or integrity of any part of the work are appropriately investigated and resolved. All persons designated as authors qualify for authorship, and all those who qualify for authorship are listed.

## CONFLICT OF INTEREST

The authors have no competing interests to disclose.

## Data Availability

All anonymised patient data is available on request.
